# Muscle weakness associated with H7N9 infection: report of two cases

**DOI:** 10.1186/s12879-018-3592-9

**Published:** 2018-12-20

**Authors:** Chao-Nan Jin, Ling-Ling Tang

**Affiliations:** 1 State Key Laboratory for Diagnosis and Treatment of Infectious Diseases, The First Affiliated Hospital, School of Medicine, Zhejiang University, Hangzhou, China; 2 Department of Infectious Disease, The State Key Laboratory for Diagnosis and Treatment of Infectious Disease, Collaborative Innovation Center for Diagnosis and Treatment of Infectious Diseases, The First Affiliated Hospital, College of Medicine, Zhejiang University, Hangzhou, 310003 China

**Keywords:** H7N9, ICU-acquired weakness, Muscle weakness, Case report, Guillain–Barré syndrome

## Abstract

**Background:**

The emerging avian influenza A (H7N9) virus, a subtype of influenza viruses, was first discovered in March 2013 in China. Infected patients frequently present with pneumonia and acute respiratory disorder syndrome with high rates of intensive care unit admission and death. Neurological complications, such as Guillain–Barré syndrome(GBS), and intensive care unit-acquired weakness, including critical illness polyneuropathy and myopathy, have only rarely been reported previously.

**Case presentation:**

In this study, we report on two Chinese patients with H7N9 severe pneumonia presenting neurological complications. These two patients had non-immune diseases prior to the onset of virus infection. A 56-year-old female patient (case 1) and a 78-year-old female patient (case 2) were admitted because of fever, cough, chest tightness and shortness of breath. These patients were confirmed to have H7N9 infection soon after admission followed by the development of acute respiratory distress syndrome and various severe bacterial and fungal infections. The case 1 patient was found to have muscle weakness in all extremities after withdrawing the mechanical ventilator, and the case 2 patient was found when withdrawing extracorporeal membrane oxygenation, both of these conditions prolonged ventilator-weaning time. Furthermore, the case 1 patient carried the H7N9 virus for a prolonged period, reaching 28 days, and both of them stayed in the hospital for more than two months. A clinical diagnosis of intensive care unit-acquired weakness could be confirmed. However, based on results from electrophysiological testing and needle electromyography of these 2 patients, it is difficult to differentiate critical illness polyneuropathy from GBS, since no lumbar puncture or muscle and nerve biopsy were conducted during hospitalization. Following a long-term comprehensive treatment, the patients’ neurological condition improved gradually.

**Conclusions:**

Although there is great improvement in saving severe patients’ lives from fatal respiratory and blood infections, it is necessary to pay sufficient attention and to use more methods to differentiate GBS from intensive care unit-acquired weakness. This unusual neurological complication could result in additional complications including ventilator associated pneumonia, prolonged hospital stay and then would further increase the death rate, and huge costs.

## Background

The first human avian influenza H7N9 cases were reported in March of 2013 in China. Most of these cases had a history of recent exposure to poultry. Associated with severe and fatal respiratory disease, avian influenza A (H7N9) virus was so fatal that many persons with confirmed H7N9 virus infection were critically ill, almost 100% of cases were hospitalized, and 34% of them died. [1] Apart from the occurrence of acute respiratory distress syndrome (ARDS) and multi-organ failure, additional atypical clinical manifestations, such as neurological presentations, were associated with fatal avian influenza infection. Additional manifestations include seizures, encephalopathy and Guillan-Barré syndrome (GBS) [2], among which GBS is usually triggered by viral or bacterial infection and characterized by acute progressive symmetric limb weakness and areflexia. GBS has been reported to result from H1N1 infection [3–5]. Considering the severe conditions of H7N9 patients, 63% [1] of whom were transferred to the intensive care unit, it is necessary to mention another neurological complication, intensive care unit-acquired weakness (ICUAW) [6–8], that frequently occurred in ICU patients. Additional neurological complications include critical illness polyneuropathy (CIP) and myopathy (CIM), the paralysis of which is typically symmetrical and affects predominantly proximal limb muscles and respiratory muscles, sparing facial and ocular muscles. In this study, we report the cases of patients infected with H7N9 who developed muscle weakness and were diagnosed with ICUAW, although we could not tell if it was CIP or GBS due to various factors.

## Case presentation

### Case 1

**Fig. 1:**
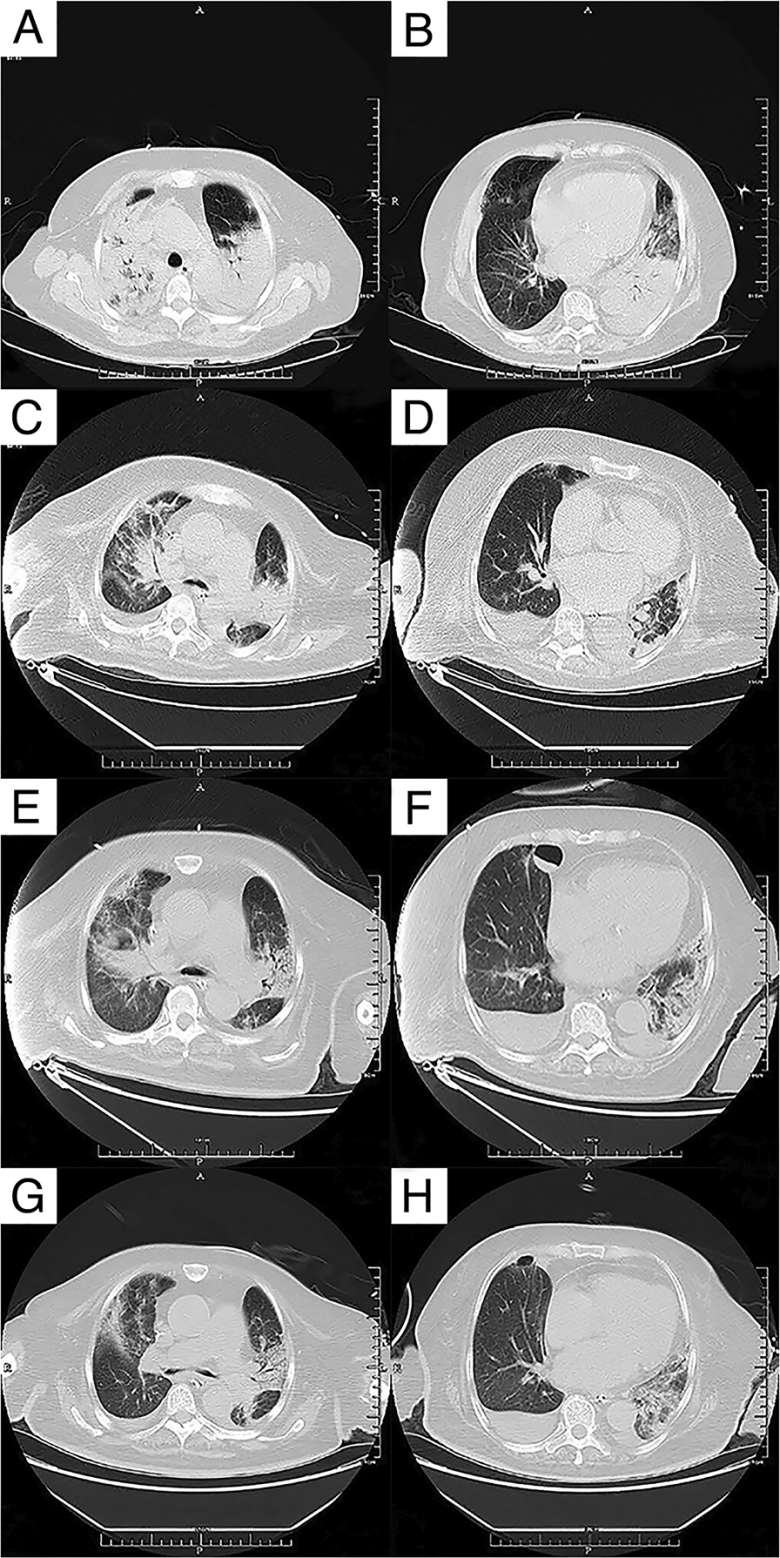
Chest computed tomography findings in Case 1*,* which were examined at the time of therapeutic initiation (**a** and **b**), 2 days before the first time withdrawing the mechanical ventilation (**c** and **d**), and in the clinical course of days 42 (**e** and **f**) and 80 (**g** and **h**)

A 78-year-old female farmer diagnosed with H7N9 infection was referred to our hospital with a 7-day history of fever (39.5 °C), cough and chest tightness. She had a well-controlled 4-year history of hypertension and reported having had contact with a dead chicken. Examination revealed stable vital signs, normal muscle strength, a white blood cell count of 6.9 × 10^9^/l, a CRP of 229 mg/L, a creatine kinase of 83 U/l, a creatine kinase-MB of 10 U/l, a lactate dehydrogenase of 278 U/l, an alanine aminotransferase of 23 U/l, and an aspartate aminotransferase of 35 U/l. A chest CT revealed the upper lobes on both sides, and the lower lobe of the left lung showed high-density and large-scale shadows. (Fig. 1a and b) She was treated with oral oseltamivir (150 mg twice), piperacillin tazobactam 4.5 g q8 h, methylprednisolone (40 mg once daily), intravenous immunoglobulin, as well as probiotics. Total parenteral nutrition was initiated and continued for 2 days with regular insulin (50 units, microinfusion pump) and was later changed to enteral nutritional suspension (500 ml daily, nasal jejunal feeding tube). By 36 h after admission, her illness rapidly progressed with the development of ARDS. Oxygen therapy and mechanical ventilation were started along with a series of ventilator-associated pneumonia(VAP) prevention strategies, such as elevating the head from the bed by 30 degrees, careful oral care, and removal of subglottic secretions.

This patient fought a severe, mixed bacterial infection throughout the next two months in the hospital and even developed septic shock on day 15. During this period, intravenous peramivir, administered 300 mg once daily, was added to the antiviral treatment on day 18, and a chest CT scan revealed a better result on day 26 (Fig. 1c and d). Another 2 days later, the tests for H7N9 virus turned negative, use of midazolam and fentanyl were stopped, and withdrawal of mechanical ventilation was attempted. Until this time, a more complicated clinical condition of limbs and respiratory muscle weakness was present, with a physical examination revealing an upper limb proximal muscle strength measurement of level 0/5, distal parts level 1/5, and lower limb muscle strength level 0/5. That of the right foot dropped significantly, and there were weak leg reflexes. Seat balance and standing balance could not be completed, which could not be explained by her improved pulmonary disease. Electrodiagnostic testing showed upper and lower limb peripheral nerve damage, which was managed with long-term neurotrophic drugs, combined with an immune-modulator, as well as physiotherapy. During this period, several attempts to withdraw the mechanical ventilation failed. A chest CT scan taken on day 42 showed that lesions had shrunk (Fig. 1e and f). Cough and other symptoms also alleviated gradually, and finally, she began absorbing oxygen via venturi mask one month later. The last chest CT scan was token on day 80 (Fig. 1g and h), and another 10 days later, she was discharged with a slight cough and expectoration and with no significant dry and wet rales heard. Her upper limb proximal muscle strength was measured at level 2/5, distal parts at level 4/5, and lower limb muscle strength at level 2/5.

### Case 2

**Fig. 2:**
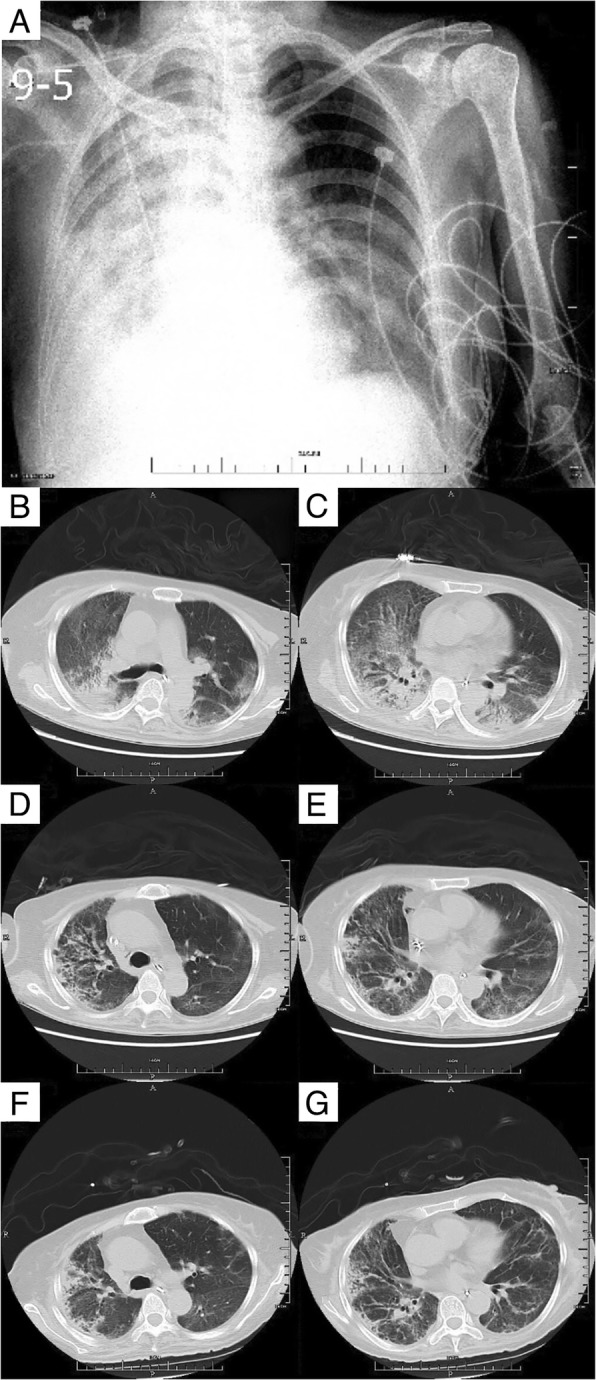
Chest X-ray and chest computed tomography findings in Case 2*,* which were examined at the time of therapeutic initiation (**a**), 5 days after tests for H7N9 virus turned negative, on the day of mechanical ventilation withdrawal (**b** and **c**), in the clinical course of day 31 (**d** and **e**) and on the day of being discharged (**f** and **g**)

A 56-year-old female patient was diagnosed with H7N9 infection and was transferred to the Emergency ICU of our hospital. She had a 3-day history fever(40 °C), cough with yellow sputum, chest tightness and shortness of breath. A chest CT scan from the local hospital showed bilateral lung infection and consolidation in part of the right lobe. She had had a resection of a meningioma brain tumour but had been well before admission. She reported having been to a live poultry market during the 2 weeks before disease onset. She had a fever (38.8 °C) and unstable vital signs when admitted. She showed a blood pressure of 79/53 mmHg and an SpO_2_ of 83% under the application of a macadamized respiring ball charged with mechanical ventilator, morphine 30 mg combined with midazolam and a combination administration of intravenous infusion via micro-pump dobutamine 100 mg, deslanoside 0.4 mg and noradrenalin 10 mg to stable her blood pressure. Another examination revealed a white blood cell count of 1.5 × 10^9^/l, a CRP of 165.90 mg/L, a creatine kinase of 199 U/l, a creatine kinase-MB of 18 U/l, a lactate dehydrogenase of 671 U/l, an alanine aminotransferase of 19 U/l, and an aspartate aminotransferase of 59 U/l. A chest X-ray (Fig. 2a) showed bilateral large dense shadows and pleural effusion. Oseltamivir (150 mg, twice daily, nasal jejunal feeding tube) and peramivir (300 mg, once daily, intravenously), as well as methylprednisolone (40 mg, once daily, microinfusion pump), piperacillin tazobactam, intravenous immunoglobulin, pinaverium bromid, and probiotics were administered on day 1. In addition, enteral nutritional suspension (500 ml daily, nasal jejunal feeding tube) was applied with regular insulin (50 units, microinfusion pump), as well as methycobal (500 μg daily, microinfusion pump) to nitrite nerves.

However, her condition persistently deteriorated. On day 5, she experienced refractory septic shock. After ineffective management of cardiopulmonary resuscitation and other rescue drugs, extracorporeal membrane oxygenation (ECMO) was initiated in combination with continuous renal replacement therapy because of anuria. Tests turned negative for H7N9 in the blood sample on day 10, then we gradually stopped using anti-viral drugs and decided to remove ECMO. 10 days later, she was found to have muscle weakness when a physical examination revealed an upper limb muscle strength measured at level 3/5 with a lower level of 2/5, which was bilateral. That of the distal parts was better than that of the proximal. When her vital signs became sufficiently stable, she received the first chest CT scan, which still revealed large dense shadows (Fig. 2b and c). Electrodiagnostic testing showed upper and lower limb peripheral nerve damage. Nasal feeding of vitamins B1 and B2 along with long-term physiotherapy, on the basis of methycobal and an immune-modulator, were initiated, as well as strategies to prevent VAP. The next two months were a long battle against severe bacterial and fungi co-infections. A chest CT scan revealed slight absorption of the lesions at the left lung on day 31(Fig. 2d and e). At last, she was discharged on day 77 after taking a chest CT scan (Fig. 2f and g) with an upper limb muscle strength measurement of level 4/5, lower limb distal of level 3/5 minus, and proximal parts of level 2/5.

## Discussion

Antiviral treatment was associated with a reduction of viral load and death rate. In this report, case 1 presented a sustained viral shedding time of 28 days, while the median has been reported to be approximately 19 days after anti-viral treatment [9, 10]. The main reason may be the late prescription of oseltamivir on day 8 in combination with peramivir on day 26 after the onset of illness, even though it has been reported that no obvious advantage is observed from antiviral treatment to viral-negative in oseltamivir-peramivir combination therapy comparing to oseltamivir monotherapy [11]. Another factor maybe associated is the application of corticosteroids, which could affect the viral shedding time on H7N9 patients [12] and may cause antiviral resistance in A/H7N9 viruses [13]. In these two cases, we prescribed methylprednisolone as 40 mg daily for 13 days and cut off to half. More solid evidence, such as randomized controlled trials, on the effect of adjuvant corticosteroids administration on viral shedding time need to be conducted on H7N9 patients.

Among 167 H7N9 infected patients admitted in our hospital centre, 89 had a severe infection and were admitted in ICU with a mean duration of mechanical ventilation of 26.3 days. Two patients, reported herein, presented a muscle weakness when we tried to withdraw mechanical ventilation, at day 28 in case 1 and at day 20 in case 2. These two patients presented bilateral and relatively symmetric flaccid weakness of the limbs with the involvement of respiratory without paralyzed cranial nerve-innervated muscles and developed decreased deep tendon reflexes, absence of an alternative diagnosis for weakness, which are consistent with Brighton Working Group clinical case definitions for GBS in level 3 certainty [14]. The diagnosis of ICUAW could be established according to the diagnosing standards [7], since features of these two critically ill patients almost matched all of risky factors of ICUAW reported before, including female, immobility, sepsis, persistent systemic inflammation, multi-organ system failure, hyperglycaemia, glucocorticoids, neuromuscular blocking agent, and bed rest [6, 15].

Furthermore, the values of CK remained in the normal range, except that upon admission, the electrophysiological examination result showed that both sensory and motor nerves were injured. This finding is more similar to CIP than CIM in ICUAW. However, electrophysiological examination enabled a diagnosis of GBS at level 1 certainty or CIP, since they both include axonal motor and sensory injury, polyneuropathy in electrophysiological feathers as part of definition. Furthermore, the condition of being on a sedative for a long time made it more difficult for observing a complete course. In addition, there are about 4 to 7 cases of influenza related GBS per 100,000 cases of influenza [16] and it has been reported that H1N1 influenza is more likely to be associated with heightened neurological complications comparing to seasonal influenza [17]. Therefore, we cannot exclude the possibility of the H7N9 virus itself contributing and resulting in paralysis roughly.

In conclusion, it is surprising, though educational, that these two mechanically ventilated patients were hospitalized for more than two months, 90 and 77 days for each, since they both experienced ARDS, severe bacterium and fungal infection, and respiratory muscle weakness. These complications resulted in ventilatory failure and, in turn, increased the possibility of VAP, which is the most common nosocomial infection in mechanically ventilated patients. There are many aspects that we can improve. Throughout the whole disease course, a series of prevention methods, such as intensive insulin therapy and passive roll over, are needed daily, as well as prevention of VAP [18].

Even though early investigation of muscle weakness is challenging, since mechanically ventilated patients are always sedated, physical examinations, especially neurological, for critically ill patients [19] are needed several times per day and should be recorded in detail. As patients gradually become alert, muscle strength should be tested in functional limb muscle groups using the Medical Research Council scale [20] or handheld dynamometry [21] to estimate the severity of force-generating impairments. This estimation is particularly important in patients with immune-mediated polyneuropathies [22], including GBS. To further differentiate between GBS and CIP, several procedures are important such as lumbar puncture, blood testing including tests for anti-GM1 antibodies, and anti-GQ1b and anti-GD1a IgG antibodies, and muscle and nerve biopsies.

## Abbreviations

ARDS: Acute respiratory distress syndrome
CIM: Critical illness myopathy
CIP: Critical illness polyneuropathy
ECMO: Extracorporeal membrane oxygenation
GBS: Guillain–Barré syndrome
ICUAW: Intensive care unit-acquired weakness
VAP: Ventilator-associated pneumonia
